# Recent Advances in Multi-Drug-Resistant Tuberculosis and RNTCP

**DOI:** 10.4103/0970-0218.43238

**Published:** 2008-10

**Authors:** Gagandeep Singh Grover, Jaspreet Takkar

**Affiliations:** District Epidemiologist, Civil Surgeon Office, Tarn Taran, Punjab, India; 1 Department of Physiology, Government Medical College, Amritsar, Punjab, India

## Introduction

Tuberculosis (TB) persists as a global public health problem of serious magnitude requiring urgent attention. Current global efforts to control TB have three distinct but overlapping dimensions: humanitarian, public health, and economic.

Alleviating illness, suffering, and death of individuals due to TB is the major humanitarian concern for a patient-centered approach to TB control. The public health dimension concerns proper diagnosis and treatment of patients with TB to decrease disease transmission. This necessitates the development of well-organized TB control programs (responsive and adaptable to the reforming health sector). TB is responsible for considerable direct and indirect costs to the individuals and the society. The economic dimension of TB control relates to a reduction of these costs, alleviation of poverty, and promotion of development.(1)

The emergence of resistance to drugs used to treat TB, and particularly multi-drug-resistant TB (MDR TB), has become a significant public health problem and an obstacle to effective TB control.(2)

Drug resistance is manifested when there is a selective growth of resistant mutants among the actively multiplying bacillary population in the presence of drugs. The emergence of drug resistance depends upon the frequency of drug resistant mutants in the susceptible bacillary population, the size of the actively multiplying bacillary population in the lesions, and the anti-microbial quality of the drugs used. Drug resistance of the Mycobacterium tuberculosis isolated from patients who have been treated for 1 month or more is defined as “acquired drug resistance”, while that of patients who have never been treated previously or treated for less than 1 month is called “primary drug resistance”.(3)

Resistance to a single drug is defined as “mono resistance” and resistance to two or more drugs is defined as “poly resistance.” Resistance to at least Isoniazid and Rifampicin is termed as “MDR”.

## Extent of the Problem

In a study among 50,000 TB cases in 35 countries, the World Health Organization (WHO), Centers for Disease Control (CDC), and International Union against Tuberculosis and Lung Diseases found that in India, Russia, Latvia, Estonia, The Dominican Republic, Argentina, and the Ivory Coast (the so called “Hot Zone”), TB was resistant to the commonly prescribed drugs Isoniazid and Rifampicin. One third of the countries surveyed had a MDR TB level between 2-14%.(4)

In another study among 64,104 TB cases from 58 geographical settings, WHO found drug resistant TB to be between 2.9% to 40.8%. The prevalence of drug resistance was directly related to the proportion of previously treated cases registered and inversely related to the proportion of TB cases treated under directly observed treatment short course (DOTS).(5)

A study conducted by the Indian Council of Medical Research (ICMR) in India in nine centers found MDR TB ranging from 0.6% to 3.2% in respect to initial drug resistance and 6% to 30% in respect to acquired drug resistance.(6)

High proportions of drug resistance have been found in Wardha, New Delhi, and Tamil Nadu. Drug resistance to Isoniazid was 20.9%, 50.7%, and 23.6% respectively while MDR TB was 9.6%, 33.7%, and 23.3%, respectively.(7)

Drug resistant TB has frequently been encountered in India and its prevalence has been known virtually from the time anti-TB drugs were introduced. However, there is no state-representated surveillance data of drug resistance among patients with TB and a major limiting factor in conducting drug resistance studies is the lack of state level Quality Assured Culture and Drug Sensitivity (DST) laboratory facilities. Tuberculosis Research Center and National Tuberculosis Institute have found MDR TB levels of less than 1% to 3% in new cases and 12% in re-treatment cases. With a rapid increase in coverage of revised national tuberculosis control programme (RNTCP) and a high cure rate observed in most regions, low emergence of drug resistance is expected across the country.(2)

## Causes of Resistance

Drug-resistant TB has microbial, clinical, and programmatic causes. From a microbiological perspective, the resistance is caused by a genetic mutation that makes a drug ineffective against the mutant bacilli. An inadequate or poorly administered treatment regimen allows drug resistant mutants to become the dominant strain in a patient infected with TB.(2)

## Transmission of Drug-Resistant TB

Drug-resistant and drug-susceptible TB is transmitted in the same way. For many years, drug-resistant TB was believed to be less infectious than drug-susceptible TB. This belief was largely based on animal studies. Now, it has been found that drug-resistant bacilli were not less infectious; in fact, contact with previously un-treated patients had a similar risk of infection, regardless of whether the bacilli were drug susceptible or drug resistant. However, an increased risk of infection has been found to occur when in contact with a patient with drug-resistant TB who had been previously treated and this increased risk resulted from prolonged exposure rather than increased infectiousness of the drug-resistant bacilli.(8)

## Prevention of MDR TB

The key to the successful prevention of the emergence of drug resistance is adequate case finding, prompt and correct diagnosis, and effective treatment of infected patients. This can be achieved through the use of DOTS.(9)

## Drug Resistance and RNTCP

A new protocol for state-wide Drug Resistance Surveillance (DRS) under RNTCP was developed in 2005. Over the next five years, RNTCP plans to systematically carry out state-wide DRS surveys in the states of Andhra Pradesh, Delhi, Gujarat, Kerala, Maharashtra, Orissa, Uttar Pradesh, and West Bengal. Besides this, the ICMR will be conducting a separate DRS in the states of Tamil Nadu and Sikkim.(10)

## DOTS Plus

DOTS Plus refers to a DOTS program that adds components for MDR TB diagnosis, management, and treatment. The WHO-endorsed DOTS Plus program began in 2000. At that time, the Green Light Committee (GLC) was established to promote access to high quality second line drugs for appropriate use in TB control programs. In 2002, the Global Fund to fight AIDS, TB, and Malaria (GFATM) started financing TB control programs, including MDR TB, greatly reducing the economic barrier to MDR TB control. DOTS-Plus programs can and should strengthen the basic DOTS strategy.(2)

## DOTS Plus and RNTCP

The RNTCP views the treatment of MDR TB patients as a ‘standard of care’ issue. Recognizing that the treatment of MDR TB cases is very complex, treatment will follow the internationally recommended DOTS Plus guidelines and will be done in designated RNTCP DOTS Plus sites. There will be at least one site in each state that will have ready access to an RNTCP-accredited culture and drug susceptibility testing (DST) laboratory.(10)

## The DOTS Plus Framework for the Management of Multi-Drug-Resistant TB

The framework is organized around the same five components of the DOTS strategy, as the underlying principles are the same. The core components are comprehensive ensuring that all essential elements of the DOTS Plus strategy are included and are as follows:

- Sustained political and administrative commitment.
  – A well-functioning DOTS program.
  – Long-term investment of staff and resources.
  – Coordination efforts between the community, local governments, and international agencies.
- Diagnosis of MDR TB through quality-assured culture and drug susceptibility testing.
  – Proper triage of patients into DST testing and the DOTS-Plus program.
- Appropriate treatment strategies that utilize second-line drugs under proper management conditions.
  – Rational treatment design (evidence-based.)
  – Directly observed therapy (DOT) ensuring long-term adherence.
  – Monitoring and management of adverse drug reactions.
- Uninterrupted supply of quality-assured anti-TB drugs.
- Recording and reporting system designed for the DOTS Plus programs that enable performance monitoring and evaluation of treatment outcome.

Each of these components involves more complex and costly operations than those for controlling drug-sensitive TB. However, addressing multi-drug resistant TB will strengthen the existing TB control program.

### Case finding strategy

At present, RNTCP does not have sufficient quality-assured laboratory capacity to do DST in all patients. Hence, the program will use a strategy that enrolls patients with a very high-risk of MDR TB into RNTCP DOTS Plus activities and treatment with the RNTCP Category IV regimen. Patients who are defined as an MDR TB suspect should be identified and investigated further for MDR TB. A MDR TB Suspect is defined as a Category II patient who is smear positive at the end of the fourth month of treatment or later.

### Drug-resistant cases

A patient is confirmed to have multi–drug-resistant TB only by an RNTCP quality assured intermediate reference laboratory (IRL). Such patients are classified according to the following definition. A confirmed MDR TB case is an MDR TB suspect who is sputum culture positive and whose TB is due to bacilli that are resistant in-vitro to at least isoniazid and rifampicin (the DST result being from an RNTCP accredited IRL).

### Bacteriology

With respect to drug-resistant TB, bacteriology includes both sputum smear microscopy and culture examination. Smear microscopy and culture should be performed and results reported according to international standards.

### Smear and culture conversion

Two separate indicators, one based on sputum smears and the other on cultures should be calculated. Patients will be considered culture converted after having two consecutive negative cultures taken at least one month apart.

## Treatment of Multi-Drug-Resistant Tuberculosis

### Classes of anti-TB drugs

The classes of anti-TB drugs have traditionally been divided into first- and second-line drugs with isoniazid, rifampicin, pyrazinamide, ethambutol, and streptomycin being the primary first-line drugs. These drugs can also be grouped based on efficacy, experience of use, and drug class. The different groups are shown in Table 1.

**Table 1:** Anti TB Drugs

Grouping	Drugs
Group 1: First line anti TB drugs	Isoniazid (H), Rifampicin (R), Ethambutol (E), Pyrazinamide (Z)
Group 2: Injectable anti TB drugs	Streptomycin (S), Amikacin (Am), Kanamycin (Km), Capreomycin (Cm),
Group 3: Fluoroquinolones	Ciprofloxacin (Cfx); Ofloxacin (Ofx); Levofloxacin (Lvx); Moxifloxacin (Mfx); Gatifloxacin (Gfx)
Group 4: Oral second-line anti-TB drugs	Ethionamide (Eto); Prothionamide anti-TB drugs (Pto); Cycloserine (Cs); Terizadone (Trd); *para*-aminosalycilic acid (PAS); Thiacetazone (T)

### Category IV regimen

RNTCP will be using a standardized treatment regimen for the treatment of MDRTB cases under the program: the Intensive Phase will consist of 6-9 months of Km, Ofx, Eto, Cs, Z, and E and the Continuation Phase will consist of 18 months of Ofx, Eto, Cs, and E.

The RNTCP will be using a standardized treatment regimen (STR), comprising of 6 drugs (kanamycin, ofloxacin, ethionamide, pyrazinamide, ethambutol, and cycloserine) during 6–9 months of the Intensive Phase and 4 drugs (ofloxacin, ethionamide, ethambutol, and cycloserine) during the 18 months of the Continuation Phase. *p*-aminosalicylic acid (PAS) is included in the regimen as a substitute drug if any of the bactericidal drugs (K, Ofl, Z, and Eto) or any 2 bacteriostatic drugs (E and Cs) are not tolerated.

### Drug dosages and administration

Drug dosages for MDR TB cases are decided according to the weight band recommendations given in Table 2.

**Table 2:** Recommended dosage according to weight in DOTS Plus

Drugs	< 45 Kg	> 45 Kg
Kanamycin	500 mg	750 mg
Ofloxacin	600 mg	800 mg
Ethionamide	500 mg	750 mg
Ethambutol	800 mg	1000 mg
Pyrazinamide	1250 mg	1500 mg
Cycloserine	500 mg	750 mg
Na PAS	10 mg	12 mg

All drugs should be given in a single daily dosage under directly observed treatment (DOT) by a DOT provider. Pyridoxine at a dose of 100mgs should be administered to all patients on an RNTCP Category IV regimen.

If a patient gains weight during treatment and crosses the weight-bands range, the DOTS Plus site committee may consider moving the patient to the higher weight-band drug dosages. The new higher dosages are provided whenever the patient is due for the next supply of drugs in the normal course of treatment and not as soon as change of weight is noted as shown in Table 3.

**Table 3:** Drug formulation and packaging in DOTS Plus

Drugs	<45 Kg	>45 Kg
Kanamycin	0.5 g vial	0.75 g vial
Ofloxacin	200 mg tablets (3)	400 mg tablets (4)
Ethionamide	250 mg tablets (2)	250 mg tablets (3)
Ethambutol	800 mg tablet (1)	1000 mg tablet (1)
Pyrazinamide	500 mg tablet (1)	750 mg tablet (2) + 750 mg tablet (1)
Cycloserine	250 mg tablets (2)	250 mg tablets (3)
Na PAS	100 g box	100 g box

In deciding about the dosages, apart from the considerations mentioned above, it is also necessary to rule out the existence of medical illnesses or organ dysfunctions in the individual by conducting routine hematological investigations like full blood count, random blood sugar, liver and kidney function tests, etc., and urine microscopy. Other investigations like skiagram, ultrasound etc. may be appropriately carried out as required in a particular case.

### Treatment duration

The recommended duration of administration of the intensive phase (IP) is guided by smear and culture conversion. The minimal recommendation is that the IP should be given for at least 6 months. After 6 months of treatment, the patient will be reviewed and the treatment changed to the CP if the culture results from the 4th month are negative. If the culture results from the 4th month remain positive, the DOTS-Plus site Committee will decide on extending the IP treatment by up to 3 months. If the 4th month culture is still awaited after 6 months of treatment, the IP will be extended until the result is available, with further treatment being decided on according to the culture result when this becomes available. After a maximum of 9 months of IP treatment, the patient will be initiated on the CP of treatment. The recommended duration for CP is 18 months.

For follow-up culture and DST, the patient needs to go to DTC. After discharge, the patient will visit the DOTS-Plus site facility only if deciding to change from the IP to the CP, at the end of treatment, at the time of the management of adverse reactions, and at the time of change of treatment due to non-response.

## Management of Contacts of MDR TB

Among contacts of patients with MDR TB, the use of isoniazid may reasonably be questioned. Close contacts of MDR TB patients should receive careful clinical follow-up for a period of at least 2 years. During this stage, no prophylactic treatment of MDR TB contacts is recommended over and above the existing RNTCP guidelines. The following measures should be taken to prevent the spread of MDR TB:

- Early diagnosis and appropriate treatment of MDR TB cases
- Screening of contacts as per RNTCP guidelines and follow-up for 2 years
- Further research into effective and non-toxic chemoprophylaxis in the areas of high MDR TB prevalence.(11–14)

## Conclusion

DOTS is a proven cost-effective TB treatment strategy. A combination of technical and managerial components, DOTS quickly makes infectious cases non-infectious and breaks the cycle of transmission. Using DOTS also prevents the development of drug-resistant strains of TB that are often fatal and very expensive to cure.(15)

Multi-drug-resistant TB is both an individual tragedy and a reflection of poor program performance. The top priority is to prevent the emergence of MDR TB by ensuring a low default rate of cases treated with first-line anti-TB drugs. If MDR TB has emerged in a certain area, it should be treated in addition to improving the basic treatment. In this situation, accurate and reliable dug susceptibility testing, methods to support patients in order to ensure direct observation of complete treatment, and the use of maximally effective regimens must be ensured. Patients with MDR TB have a good chance for a cure with second-line drugs, hence the treatment, if it is to be provided, should be optimally selected and administered.(16) Second-line drugs should not be kept in reserve and the treatment observation must be ensured.
